# Long-term effects of difference dosage of pulmonary rehabilitation program in post severe COVID-19 patients

**DOI:** 10.3389/fmed.2025.1649667

**Published:** 2025-10-07

**Authors:** Rodrigo Muñoz-Cofré, Jorge Valenzuela, Constanza Díaz Canales, Máximo Escobar-Cabello, Fernando Valenzuela-Aedo, Daniel Conei, Rodrigo Lizama-Pérez, Gabriel Nasri Marzuca-Nassr, Mariano del Sol, María Fernanda del Valle

**Affiliations:** ^1^Centro de Excelencia en Estudios Morfológicos y Quirúrgicos (CEMyQ), Facultad de Medicina, Universidadde La Frontera, Temuco, Chile; ^2^Departamento de Especialidades Médicas, Facultad de Medicina, Universidad de La Frontera, Temuco, Chile; ^3^Servicio de Medicina Física y Rehabilitación, Hospital El Carmen, Maipú, Chile; ^4^Laboratorio de Función Disfunción ventilatoria, Departamento de Kinesiología, Universidad Católica del Maule, Talca, Chile; ^5^Escuela de Kinesiología, Facultad de Salud, Universidad Santo Tomás, Temuco, Chile; ^6^Departamento de Ciencias de la Rehabilitación, Facultad de Medicina, Universidad de La Frontera, Temuco, Chile; ^7^Doctorado en Ciencias Morfológiccas, Facultad de Medicina, Universidad de La Frontera, Temuco, Chile; ^8^Departamento de Procesos Terapéuticos, Facultad de Ciencias de la Salud, Universidad Católica de Temuco, Temuco, Chile; ^9^Departamento de Anatomía Normal y Medicina Legal, Facultad de Medicina, Universidad de Concepción, Concepción, Chile; ^10^Departamento de Ciencias Morfológicas, Facultad de Medicina y Ciencia, Universidad San Sebastián, Concepción, Chile

**Keywords:** COVID-19, exercise performance, pulmonary function, pulmonary rehabilitation, quality of life

## Abstract

**Background:**

COVID-19 can lead to severe respiratory complications requiring invasive mechanical ventilation (IMV). Post-acute sequelae often include reduced pulmonary function, decreased physical capacity, and impaired quality of life. Pulmonary rehabilitation programs (PRPs) have shown promise in aiding recovery, but the long-term effectiveness and optimal dosage (number of sessions) remain unclear.

**Methods:**

A experimental, repeated-measures study was conducted at Hospital El Carmen de Maipú, Chile, involving 60 adults (male and female) who had received IMV due to severe COVID-19. Participants completed an individualized PRP consisting of sessions held twice weekly. Each session included 30 minutes of aerobic exercise, 20 minutes of strength training, and 10 minutes of stretching exercises. Participants were assigned to one of three intervention arms: 12, 24, or 36 sessions. Clinical outcomes included spirometric parameters, 6-min walk distance (6-MWD), Hand Grip Strength (HGS), functional status, and dyspnea. Psychological outcomes included quality of life and fatigue. Assessments were conducted at baseline, post-intervention, and 1 year after the intervention.

**Results:**

Twelve-session group, significant improvements in Maximum Inspiratory Pressure (MIP) and 6-MWD were observed (p < 0.05). Clinical and psychological improvements were sustained at 1 year. Twenty four-session group, significant changes were found in Forced Vital Capacity (FVC % predicted) and right-hand grip strength (HGS) (*p* < 0.05). Improvements in clinical and psychological variables persisted at 1 year, though additional gains were observed only in spirometric parameters between post-intervention and follow-up. Thirty six-session group, participants experienced significant improvements in physical and mental Fatigue Assessment Scale (FAS) scores, total FAS, and bodily pain (*p* < 0.05). These benefits remained stable at the 1-year evaluation, with no significant changes between post-intervention and follow-up.

**Conclusion:**

Individualized PRPs produced significant improvements in clinical and psychological outcomes in patients recovering from severe COVID-19 requiring IMV. Importantly, these benefits were maintained 1 year after the intervention, regardless of the number of sessions (12, 24, or 36). The lack of significant long-term differences among groups suggests that a shorter but personalized rehabilitation program may be sufficient to produce durable improvements in this population. These findings support the implementation of tailored PRPs as a key component of post-COVID-19 care.

## Introduction

In December 2019, the World Health Organization (WHO) issued a warning about individuals in Wuhan, China, who were suffering from pneumonia of unknown etiology. This would be the initial focus of a pandemic that affected the entire world (1).

In Chile, as of May 13, 2021, 1,512,239 cases of COVID-19 had occurred, with a rate of 7,771.7 per 100,000 inhabitants (2). Although most of these cases were treated and survived, the projections of the functional consequences this group of patients will present in the future are still a matter of speculation (3, 4).

The primary feature of the clinical picture was the emergence of swiftly evolving respiratory symptoms, which, in certain instances, could result in acute respiratory failure. In this context, a patient group with COVID-19 required prolonged invasive mechanical ventilation (IMV) (5). This condition, when combined with extended bed rest, results in a range of sequelae, such as dyspnea and decreased muscle mass and strength, which have a detrimental impact on the functional capacity of these patients (6–8).

Given these circumstances, it is imperative to establish pulmonary rehabilitation programs (PRP) during and after hospitalization (9). PRP require a comprehensive individualized assessment including pulmonary function, aerobic capacity, and strength testing (6–9). Previous reports from this research group have demonstrated positive short-term outcomes of PRP performed over 12, 24, and 36 sessions (6–18 weeks) in patients with COVID-19 who required IMV, observing a significant increase in forced vital capacity (FVC), distance traveled on the 6-min walk distance (6-MWD), and handgrip strength (HGS) as well as a significant decrease in perceived fatigue and dyspnea (7, 10, 11).

The long-term effects of severe COVID-19 remain under investigation, with preliminary data pointing to potential similarities with acute respiratory distress syndrome (ARDS) (12–15). Thus, if questions remain regarding long-term sequelae of COVID-19, it is also reasonable to ask whether the effects of a PRP will be sustained over time. These data could have repercussions on the progressive increase in demands for health care services, where recovery of the functionality of these patients should be the central focus of comprehensive care (16).

Therefore, this study assessed the long-term effects (~1 year) of an individualized PRP (12 vs. 24 vs. 36 sessions) in patients with COVID-19 connected to IMV.

## Materials and methods

### Participants

**Figure 1:**
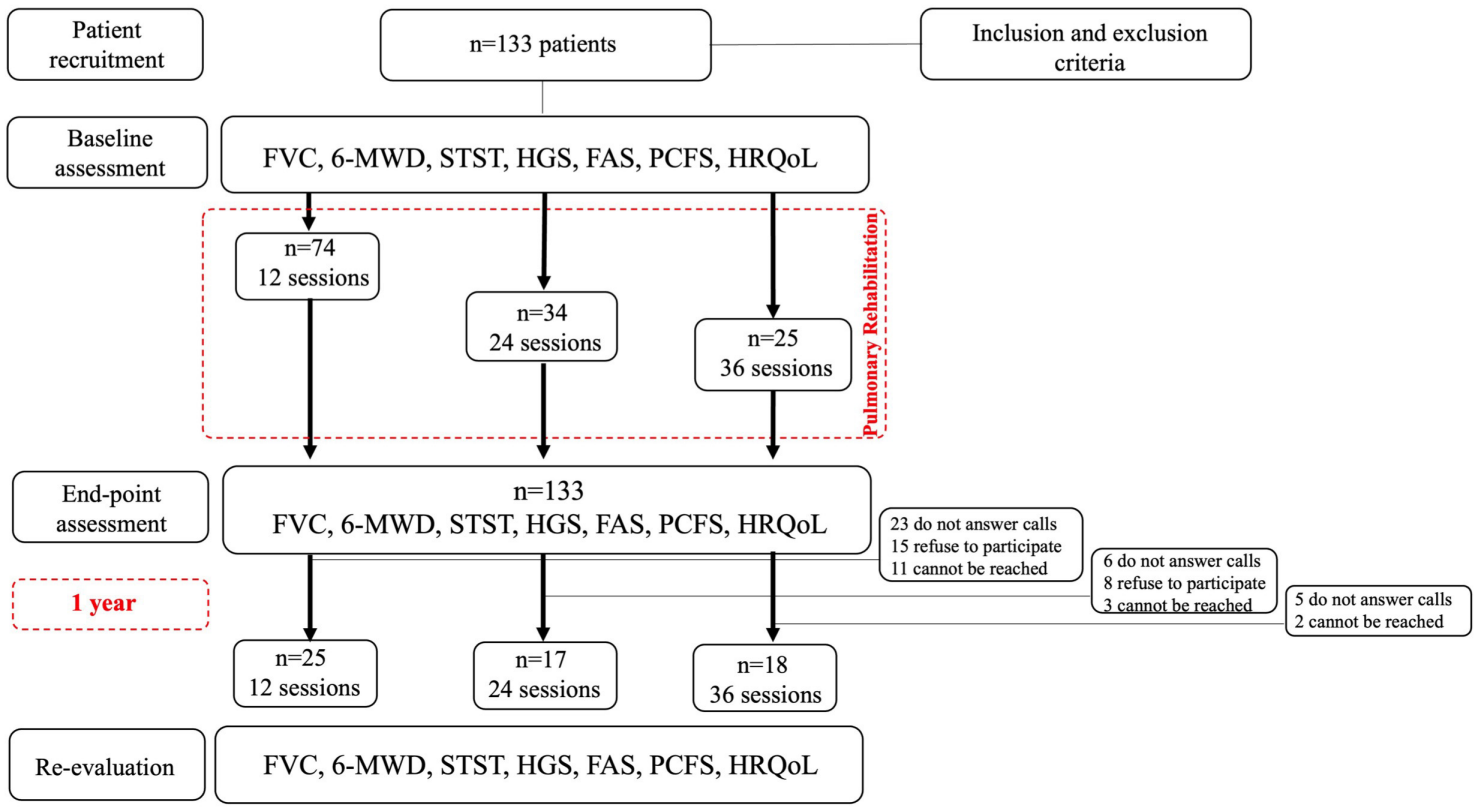
Flowchat. N, number of participants; FVC, forced vital capacity; 6-MWD, 6-min walk distance; STST, sit-to-stand test; HGS, hand-grip strength; FAS, fatigue assessment scale; PCFS, post-COVID-19 functional status; HRQoL, health-related quality of life.

In this experimental repeated measures study, sixty patients were included (Figure 1). Non-probability and consecutive sampling was used. The study was conducted between September 2020 and September 2022. This study was approved by the Scientific Ethics Committee of the Central Metropolitan Health Service of Chile (Resolution No. 378/2021). This project has previous publications that provide preliminary results or secondary analyses (4, 7, 10, 11, 17). All participants were informed about the procedures of this study, agreed to participate, and gave their written consent. Inclusion criteria were (a) having gone through the PRP at Hospital El Carmen (HEC), (b) having completed the PRP, and (c) having attended follow-ups with a bronchopulmonary specialist. At the completion of the PRP, patients were allocated into three groups based on the total number of sessions completed: 12s Group (12 sessions), 24s Group (24 sessions), and 36s Group (36 sessions). The classification was determined by assessing each patient's ability to walk continuously for 30 minutes on a treadmill at the end of the initial 12 sessions. Patients who achieved this target concluded their PRP at that point. Those who did not meet the criterion continued until completing 24 sessions, at which time they were re-evaluated. Following the same criterion, patients either concluded the program after 24 sessions or proceeded until the completion of 36 sessions (17).

### Measurements

The evaluations were carried out in 3 phases: (i) the PRP was carried out between September 2020 and September 2021, (ii) after the PRP was completed, the re-evaluation was carried out, and (iii) between September 2021 and September 2022, the 1-year follow-up re-evaluation was conducted. The measurements performed were as follows:

**Spirometry:** A Medgraphics spirometer (CPFS/D USB 2.02, MGC Diagnostics Corporation, Minnesota, USA) was used. The following parameters were evaluated: FVC, forced expiratory volume in the first second (FEV_1_), and the ratio between the two, following the current ATS/ERS recommendations (FEV_1_/FVC) (18).

**Maximum inspiratory pressure (MIP):** A differential pressure gauge (PCE-P01/PCE-P05^®^, PCE Ibérica S.L. Albacete, Spain) was used for this purpose. The MIP measurement was standardized according to ATS/ERS standards (19).

**Aerobic capacity:** The 6-MWD was conducted in a 30-meter-long, traffic-free corridor. Patients were instructed that the object of the test was to walk as many meters as possible for 6 min (20). Dyspnea and lower limb fatigue were categorized with the modified Borg scale (21). Pulse oximetry was measured at the beginning and end of the 6-MWD with a pulse oximeter (Nonin 7500^®^, Nonin Medical, Minnesota, USA). The distance covered was recorded in meters.

**Lower limb strength:** This was evaluated using the sit-to-stand test (STST). The patient was seated in a chair with their arms crossed and close to their chest. At the command “ready go”, they had to stand up and sit down the greatest number of times in 1 minute (22). The number of repetitions achieved was recorded.

**Grip strength:** The maximum Hand Grip Strength (HGS) was performed with a hydraulic dynamometer (Jamar^®^, Missouri, USA). In this evaluation, the patient must exert maximum pressure for 3 s, with 1 min of rest between each repetition, performing two attempts, where the best of the two attempts was used for the study (23).

**Fatigue:** This was measured with the Fatigue Assessment Scale (FAS). The FAS questionnaire is self-administered and includes dimensions of physical and mental fatigue. The response scale is a 5-point Likert-type scale, where 1: never; 2: sometimes (approximately monthly or less); 3: regularly (a few times a month); 4: often (approximately weekly); 5. always (approximately every day) (24).

**Post-COVID-19 functional status (PCFS):** This is a scale with four questions to which the following progression is assigned: grade 0: no functional limitations; grade 1: very mild functional limitations; grade 2: mild functional limitations; grade 3: moderate functional limitations; and grade 4: severe functional limitations. Functional limitation is assigned in relation to the last week (exception: when evaluated at hospital discharge, it is the situation on the day of discharge). Symptoms reported are dyspnea, pain, fatigue, muscle weakness, memory loss, depression, and anxiety. If questions have the same degree of functional limitation, the question with the highest degree of limitation is selected (25).

**Health-related quality of life:** Health-related quality of life (HRQoL) was assessed using Version 2 of the Short-Form 36 Health Survey (SF-36), which is an instrument developed in the United States to assess HRQoL in adults (Ware and Sherbourne, 1992). The SF-36 has been adapted syntactically and semantically for the Chilean population (26). The questionnaire includes 36 items measured on a Likert scale. The 36 items are grouped into 8 health topics: physical functioning (PF), physical role (PR), bodily pain (BP), perception of general health (GH), vitality (VT), social functioning (SF), emotional role (ER), and mental health (MH).

### Intervention

**Pulmonary Rehabilitation Program:** The sessions (twice a week) were divided into 30 min of aerobic exercise, 20 min of strength exercise, and 10 min of flexibility consisting of muscle stretching. The training session was stopped when the participant presented one of the following criteria: dyspnea or fatigue ≥7 points (out of 10), a pulse saturation < 91%, or exceeded 80% of their heart rate reserve. In addition, inspiratory muscle strength training was performed at each patient's home (twice a day, 5 times a week). The PRP is detailed in previous publications (7, 10).

### Statistical analysis

The statistical power of the recruited sample was determined with the G^*^power 3.1.9.7 software, specifically, an F test with one-way ANOVA was used, considering the mean FVC and the number of participants per group. The normality of the data was assessed using the Shapiro–Wilk test. Data are presented as mean ± standard deviation along with minimum and maximum values. Initial, final, and one-year re-evaluations data are presented as mean ± standard deviation and were analyzed using repeated-measures ANOVA or the Kruskal–Wallis test, as appropriate, with time as the within-subjects factor and 12s, 24s, and 36s group as the between-subjects factor. In the case of a significant interaction, Bonferroni post hoc tests were conducted when necessary. A significance level of α = 0.05 was adopted. Statistical analyses were performed using SPSS v.24.0 (IBM Corp., Armonk, NY, USA).

## Results

In the 12s group, 25 patients were included, 49 did not participate because: 23 did not answer calls 15 refused to participate 11 could not be reached. In the 24s group, 17 patients were included, 17 did not participate because: 6 did not answer calls 8 refused to participate 3 could not be reached. In the 36s group, 18 patients were included, 7 did not participate because: 5 did not answer calls 2 could not be reached (Figure 1). The statistical power (1-β err prob) of the sample analyzed was 0.67.

### Baseline characteristics

Table 1 presents the baseline characteristics of the participants when compared by group (12, 24, and 36 sessions). Regarding spirometric parameters, significant differences were observed in the absolute value of FVC, which was significantly higher in the 12s group compared to the 24s group (*p* = 0.045). Similarly, FEV_1_ was significantly higher in the 12s group compared to the 36s group (*p* = 0.050). In terms of physical condition, the 6-MWD was significantly higher in the 12s group compared to the 36s group (*p* = 0.001), and the number of repetitions in the STST was also significantly higher in the 12s group compared to the 36s group (*p* = 0.006). With respect to fatigue, functionality, and dyspnea, the physical FAS score was significantly lower in the 12s group compared to the 24s (*p* = 0.003) and 36s (*p* = 0.043) groups. The total FAS score was significantly lower in the 12s group compared to the 24s group (*p* = 0.01). In terms of functionality, the PCFS score was significantly lower in the 12s group compared to the 36s group (*p* = 0.034). For dyspnea, the mMRC score was significantly lower in the 12s group compared to the 24s (*p* = 0.019) and 36s (*p* = 0.046) groups; in addition, the Borg scale score was significantly lower in the 12s group compared to the 24s group (*p* = 0.001). Regarding HRQoL, the RE item score was significantly higher in the 36s group (*p* = 0.023). Finally, concerning medical history, the prevalence of diabetes mellitus was significantly higher in the 12s group (17/68.00%, *p* = 0.040) (Table 1).

**Table 1:** Comparison of the baseline assessment of participants, divided into groups according to number of sessions.

**Variable**	**12s Group**	**24s Group**	**36s Group**	***p*-value**
**General characteristics**				
*N*/percentage (Female/Male)	25/42% (9/16)	17/28% (6/11)	18/30% (6/12)	0.984
Age (years)	58 ± 13 (18.00–77.00)	62 ± 7 (51.00–77.00)	56 ± 10 (44.00–77.00)	0.317
Weight (kg)	83.53 ± 15.64 (55.00–111.00)	84.58 ± 12.71 (68.50–110.00)	82.32 ± 16.69 (51.20–122.00)	0.908
Height (m)	1.63 ± 0.07 (1.46–1.74)	1.64 ± 0.07 (1.50–1.79)	1.63 ± 0.07 (1.45–1.74)	0.779
BMI (kg/m^2^)	31.57 ± 6.18 (21.21–44.07)	31.41 ± 5.42 (24.22–31.41)	31.12 ± 7.59 (17.71–47.65)	0.975
IMV (days)	20.68 ± 12.28 (3.00–43.00)	27.06 ± 21.08 (7.00–75.00)	35.94 ± 32.10 (4.00–138.00)	0.094
Prone (days)	3.40 ± 2.27 (0–6.00)	4.24 ± 3.68 (0–12.00)	5.72 ± 4.71 (0–12.00)	0.115
Worker (Yes/No)	0/25	0/17	0/18	–
**Spirometrics parameters**				
FVC (L)	3.38 ± 0.77 (1.74–5.03)	2.71 ± 0.85 (1.38–4.33)	2.75 ± 0.75 (1.46–4.00)	**0.045** ^**#**^
FVC (%Pred)	93.16 ± 16.67 (59.00–121.00)	77.35 ± 22.25(45.00–116.00)	74.17 ± 20.88 (36.00–107.00)	**0.040**^**#**^ 0.008^*^
FEV_1_ (L)	2.73 ± 0.64 (1.47–4.15)	2.27 ± 0.74 (0.85–3.47)	2.22 ± 0.69 (0.40–3.31)	**0.050** ^*^
FEV_1_ (%Pred)	96,68 ± 17.95 (61.00–123.00)	83.06 ± 20.93 (39.00–115.00)	76.78 ± 25.22 (12.00–113.00)	**0.011** ^*^
FEV_1_/FVC (%)	81.84 ± 4.38 (72.00–91.00)	82.59 ± 7.00 (62.00–90.00)	86.44 ± 8.84 (57.00–100.00)	0.080
MIP (-cmH_2_O)	65.79 ± 23.71 (17.60–114.00)	58.01 ± 31.30 (13.20–123.50)	62.14 ± 31.03 (14.00–143.00)	0.625
**Physical condition**				
6-MWD (m)	453.48 ± 90.38 (276.00–574.00)	386.18 ±- 133.83 (180.00–582.00)	318.11 ± 114.68 (150.00–575.00)	**0.001** ^*^
STST (rpm)	21.91 ± 5.36 (7.00–32.00)	19.00 ± 6.74 (2.00–31.00)	15.22 ± 8.27 (0–34.00)	**0.006** ^*^
HGS right (kg)	18.96 ± 8.48 (0–40.00)	22.53 ± 15.93 (2.00–60.00)	20.94 ± 18.59 (0–62.00)	0.725
HGS left (kg)	16.56 ± 11.91 (0–50.00)	19.65 ± 15.82 (2.00–60.00)	17.33 ± 17.43 (0–60.00)	0.799
**Fatigue, Functional status and dyspnea**				
Physical FAS (points)	11.28 ± 3.70 (5.00–20.00)	15.53 ± 3.41 (8.00–21.00)	14.33 ± 4.58 (7.00–22.00)	**0.003**^**#**^ 0.043^*^
Mental FAS (points)	10.28 ± 4.75 (5.00–21.00)	13.18 ± 4.85 (5.00–22.00)	11.11 ± 3.74 (6.00–19.00)	0.129
Total FAS (points)	21.56 ± 7.76 (11.00–39.00)	28.53 ± 6.50 (15.00–42.00)	25.44 ± 7.42 (13.00–39.00)	**0.01** ^**#**^
PCFS (points)	2.56 ± 0.96 (1.00–4.00)	2.94 ± 0.96 (2.00–4.00)	3.22 ± 0.87 (1.00–4.00)	**0.034** ^*^
mMRC (points)	1.36 ± 1.03 (0–3.00)	2.35 ± 1.05 (1.00–4.00)	2.22 ± 1.26 (0–4.00)	**0.019**^**#**^ 0.046^*^
Borg (points)	0.12 ± 0.44 (0–2.00)	1.97 ± 2.25 (0–9.00)	1.00 ± 1.13 (0–4.00)	**0.001** ^**#**^
**Health-related quality of life**				
Physical functioning	52.40 ± 27.58 (5.00–95.00)	67.35 ± 26.58 (10.00–100.00)	36.94 ± 22.23 (0–80.00)	0.143
Role physical	23.00 ± 38.81 (0–100.00)	41.18 ± 41.40 (0–100.00)	8.33 ± 14.85 (0–50.00)	0.232
Bodily pain	43.40 ± 28.15 (0–100.00)	56.73 ± 24.50 (12.50–100.00)	36.80 ± 32.54 (0–100.00)	0.734
General health	51.60 ± 17.89(15.00–95.00)	58.82 ± 26.78 (10.00–100.00)	47.78 ± 18.32 (5.00–90.00)	0.327
Vitality	52.00 ± 25.24 (0–100.00)	64.12 ± 22.51 (25.00–100.00)	37.50 ± 26.80 (0–85.00)	0.134
Social functioning	66.08 ± 22.09 (28.00–100.00)	69.61 ± 26.27 (12.50–100.00)	60.41 ± 36.44 (0–100.00)	0.319
Role emotional	50.66 ± 44.22 (0–100.00)	56.86 ± 49.67 (0–100.00)	66.67 ± 48.50 (0–100.00)	**0.023** ^**&**^
Mental health	36.00 ± 25.08 (0–100.00)	66.18 ± 30.54 (25.00–100.00)	23.61 ± 30.28 (0–100.00)	0.327
**Medical History**				
Obesity (*n*/%)	14 (56.00%)	8 (47.00%)	8 (44.44%)	0.641
HBP (*n*/%)	16 (64.00%)	14 (82.35%)	10 (55.55%)	0.244
DM (*n*/%)	17 (68.00%)	8 (47 %)	6 (33.33%)	**0.040** ^*^

Data are presented as mean ± standard deviation (minimum-maximum).; *N*, number of participants; BMI, body mass index; IMV, invasive mechanical ventilation; FVC, forced vital capacity; FEV_1_, forced expiratory volume in the first second; MIP, maximum inspiratory pressure; cmH_2_O, centimeters of water; 6-MWD, 6-min walk distance; STST, sit-to-stand test; HGS, hand-grip strength; FAS, fatigue assessment scale; PCFS, post-COVID-19 functional status; mMRC, modified Medical Research Council; Borg, modified Borg scale; %, percentage; DM, diabetes mellitus; HBP, high blood pressure; Bold values denote *p* < 0.05; post hoc #, 12s vs. 24 s; ^*^, 12s vs. 36s; &, 24s vs. 36s.

### Pulmonary rehabilitation, intragroup analysis

The results for the pre-, post-, and 1-year measurements for the 12s, 24s, and 36s groups are shown in Tables 2–4, respectively.

**Table 2:** Comparison of initial, final, and year-end re-evaluations in participants of the 12-session pulmonary rehabilitation group (*n* = 25).

**Variable**	**Pre PRP**	**Post PRP**	**1-year re-evaluation**	**ANOVA *p* value**
**Spirometrics parameters**				
FVC (L)	3.38 ± 0.77 (1.74–5.03)	3.43 ± 0.80 (1.84–4.98)	3.47 ± 0.82 (1.65–5.07)	0.97
FVC (%Pred)	93.16 ± 16.67 (59.00–121.00)	95.58 ± 14.93 (62.00–124.00)	96.80 ± 15.95 (63.00–130.00)	0.10
FEV_1_ (L)	2.73 ± 0.64 (1.47–4.15)	2.73 ± 0.70 (1.49–4.09)	2.85 ± 0.67 (1.41–4.15)	0.11
FEV_1_ (%Pred)	96,68 ± 17.95 (61.00–123.00)	100.08 ± 17.35 (59.00–126)	101.52 ± 17.51 (65.00–132.00)	**0.042** ^**#**^
FEV_1/_FVC (%)	81.84 ± 4.38 (72.00–91.00)	81.88 ± 4.54 (74.00–90.00)	82.28 ± 4.07 (57.00–100.00)	0.71
MIP (-cmH_2_O)	67.60 ± 27.01 (17.60–114.00)	79.40 ± 24.93 (35.00–160.00)	73.88 ± 19.03 (39.00–111.10)	**0.001** ^**#**^
**Physical condition**				
6-MWD (m)	453.48 ± 90.38 (276.00–574.00)	520.72 ± 90.09 (338.00–664.00)	516.72 ± 94.35 (275.00–620.00)	**0.001** ^#, *^
STST (rpm)	21.91 ± 5.36 (7.00–32.00)	25.96 ± 5.18 (16.00–38.00)	26.96 ± 6.92 (12.00–41.00)	**0.001** ^#, *^
HGS right (kg)	18.96 ± 8.48 (0–40.00)	23.48 ± 9.72 (5.00–46.00)	25.44 ± 8.13 (10.00–40.00)	**0.001**^**#**^ 0.007^*^
HGS left (kg)	16.56 ± 11.91 (0–50.00)	21.04 ± 13.56 (0–60.00)	23.60 ± 7.62 (9.00–35)	**0.001**^**#**^ 0.005^*^
**Fatigue, Functional status and dyspnea**				
Physical FAS (points)	11.28 ± 3.70 (5.00–20.00)	9.16 ± 3.79 (5.00–19.00)	8.40 ± 3.45 (5.00–18.00)	**0.001** ^#, *, &^
Mental FAS (points)	10.28 ± 4.75 (5.00–21.00)	7.68 ± 2.79 (5.00–16.00)	7.72 ± 3.06 (5.00–19.00)	**0.003**^**#**^ 0.027^*^
Total FAS (points)	21.56 ± 7.76 (11.00–39.00)	17.20 ± 6.92 (10.00–39.00)	16.12 ± 6.16 (10.00–36.00)	**0.001** ^#, *^
PCFS (points)	2.56 ± 0.96 (1.00–4.00)	1.04 ± 1.13 (0–4.00)	0.68 ± 0.74 (0–2.00)	**0.001** ^#, *^
mMRC (points)	1.36 ± 1.03 (0–3.00)	0.36 ± 0.70 (0–2.00)	0.28 ± 0.61 (0–2.00)	**0.001** ^#, *^
Borg (points)	0.12 ± 0.44 (0–2.00)	0.08 ± 0.40 (0–2.00)	0.12 ± 0.33 (0–1.00)	0.844
**Health-related quality of life**				
Physical functioning	52.40 ± 27.58 (5.00–95.00)	69.20 ± 29.64 (10.00–100.00)	79.80 ± 20.69 (20.00–100.00)	**0.004**^**#**^ 0.001^*^
Role physical	23.00 ± 38.81 (0–100.00)	52.00 ± 45.02 (0–100.00)	67.00 ± 44.32 (0–100.00)	**0.01**^**#**^ 0.001^*^
Bodily pain	43.40 ± 28.15 (0–100.00)	56.58 ± 28.64 (0–100.00)	71.56 ± 23.53 (25.00–100.00)	**0.049**^**#**^ 0.006^*^
General health	51.60 ± 17.89 (15.00–95.00)	63.00 ± 25.90 (15.00–100.00)	62.71 ± 19.44 (15.00–95.00)	0.087
Vitality	52.00 ± 25.24 (0–100.00)	66.60 ± 28.38 (0–100.00)	75.21 ± 20.02 (30.00–100.00)	**0.012**^**#**^ 0.006^*^
Social functioning	66.08 ± 22.09 (28.00–100.00)	71.24 ± 26.59 (4.00–100.00)	80.67 ± 16.95 (48.00–100.00)	**0.026**^**#**^ 0.003^*^
Role emotional	50.66 ± 44.22 (0–100.00)	65.66 ± 46.66 (0–100.00)	75.00 ± 41.99 (0–100.00)	0.146
Mental health	36.00 ± 25.08 (0–100.00)	54.20 ± 34.51 (0–100.00)	80.21 ± 23.28 (0–100.00)	**0.023**^**#**^ 0.001^*^0.037^**&**^

Data are presented as mean ± standard deviation (minimum-maximum). Pre RPR, before the pulmonary rehabilitation program; Post PRP, after the pulmonary rehabilitation program; FVC, forced vital capacity; FEV_1_, forced expiratory volume in the first second; MIP, maximum inspiratory pressure; cmH_2_O, centimeters of water; 6-MWD, 6-min walk test; STST, sit-to-stand test; HGS, hand-grip strength; FAS, fatigue assessment scale; PCFS, post-COVID-19 functional status; mMRC, modified Medical Research Council; Borg, modified Borg scale. Bold values denote *p* < 0.05; post hoc #, Pre vs. Post; ^*^, pre vs. 1-year re-evaluation; &, post vs. 1-year re-evaluation.

**Table 3:** Comparison of initial, final, and one-year re-evaluations in participants of the 24-session pulmonary rehabilitation group (*n* = 17).

**Variable**	**Pre PRP**	**Post PRP**	**1-year re-evaluation**	**ANOVA *p* value**
**Spirometrics parameters**				
FVC (L)	2.71 ± 0.85 (1.38–4.33)	2.90 ± 0.83 (1.43–4.24)	3.05 ± 0.83 (1.51–4.31)	**0.05** ^**#**^
FVC (%Pred)	77.35 ± 22.24 (45.00–116.00)	81.65 ± 19.40 (53.00–116.00)	87.38 ± 17.62 (57.00–124.00)	**0.030**^**#**^ 0.040^*^
FEV_1_ (L)	2.27 ± 0.74 (0.85–3.47)	2.36 ± 0.90 (0.90–3.62)	2.53 ± 0.76 (1.00–3.72)	**0.036**
FEV_1_ (%Pred)	83.06 ± 20.93 (39.00–115.00)	84.24 ± 19.94 (42.00–115.00)	92.31 ± 19.61 (48.00–130.00)	**0.011** ^**&**^
FEV1/FVC (%)	82.59 ± 7.00 (62.00–90.00)	80.59 ± 6.49 (63.00–91.00)	82.06 ± 5.97 (66.00–90.00)	**0.011**^**#**^ **0.012**^**&**^
MIP (-cmH_2_O)	58.01 ± 31.30 (13.20–123.50)	74.43 ± 38.32 (24.30–157.60)	71.85 ± 26.50 (27.80–109.90)	**0.001** ^**#**^
**Physical condition**				
6-MWD (m)	386.18 ± 133.83 (180.00–562.00)	468.00 ±- 113.23 (259.00–621.00)	481.07 ± 105.22 (193.00–603.00)	**0.001**^**#**^ 0.006^*^
STST (rpm)	19.00 ± 6.74 (2.00–31.00)	25.06 ± 7.04 (8.00–35.00)	25.21 ± 5.87 (8.00–33.00)	**0.001** ^#, *^
HGS right (kg)	22.53 ± 15.93 (2.00–60.00)	29.82 ± 20.85 (4.00–78.00)	22.19 ± 11.32 (5.00–41.00)	**0.01** ^**#**^
HGS left (kg)	19.65 ± 15.82 (2.00–60.00)	25.82 ± 22.14 (5.00–78.00)	19.69 ± 11.31 (0–37.00)	**0.048** ^**#**^
**Fatigue, Functional status and dyspnea**				
Physical FAS (points)	15.53 ± 3.41 (8.00–21.00)	12.53 ± 5.26 (5.00–22.00)	10.53 ± 4.71 (6.00–19.00)	**0.001** ^*^
Mental FAS (points)	13.18 ± 4.85 (5.00–22.00)	10.71 ± 4.08 (5.00–17.00)	9.88 ± 4.35 (5.00–18.00)	**0.026** ^*^
Total FAS (points)	28.53 ± 6.50 (15.00–42.00)	23.24 ± 8.66 (10.00–34.00)	20.41 ± 8.39 (11.00–37.00)	**0.002** ^*^
PCFS (points)	2.94 ± 0.96 (2.00–4.00)	1.41 ± 1.17 (0–3.00)	1.35 ± 0.70 (0–3.00)	**0.001**^**#**^,^*^
mMRC (points)	2.35 ± 1.05 (1.00–4.00)	0.65 ± 0.93 (0–3.00)	0.53 ± 0.80 (0–3.00)	**0.001**^**#**^,^*^
Borg (points)	1.97 ± 2.25 (0–9.00)	0.18 ± 0.52 (0–2.00)	0.29 ± 0.77 (0–3.00)	**0.016**^**#**^ 0.023^*^
**Health-related quality of life**				
Physical functioning	45.00 ± 23.51 (0–80.00)	67.35 ± 26.58 (10.00–100.00)	75.63 ± 25.55 (0–100.00)	**0.039# 0.004** ^*^
Role physical	26.47 ± 39.00 (0–100.00)	41.18 ± 41.40 (0–100.00)	40.63 ± 36.37 (0–100.00)	0.304
Bodily pain	40.88 ± 17.13 (10.00–67.50)	56.73 ± 24.50 (12.50–100.00)	47.18 ± 21.86 (10.00–80.00)	0.062
General health	44.71 ± 18.66 (10.00–80.00)	58.82 ± 26.78 (10.00–100.00)	48.75 ± 21.79 (5.00–90.00)	0.093
Social functioning	43.38 ± 33.10 (0–100.00)	69.61 ± 26.27 (12.50–100.00)	69.53 ± 31.94 (12.50–100.00)	**0.011**^**#**^ 0.042^*^
Role emotional	25.48 ± 38.23 (0–100.00)	56.86 ± 49.67 (0–100.00)	47.91 ± 47.09 (0–100.00)	0.065
Mental health	30.88 ± 24.25 (0–75.00)	66.18 ± 30.54 (25.00–100.00)	71.88 ± 23.93 (25.00–100.00)	**0.001** ^#, *^

Data are presented as mean ± standard deviation (minimum-maximum). Pre RPR, before the pulmonary rehabilitation program; Post PRP, after the pulmonary rehabilitation program; FVC, forced vital capacity; FEV_1_, forced expiratory volume in the first second; MIP, maximum inspiratory pressure; cmH_2_O, centimeters of water; 6-MWD, 6-min walk test; STST, sit-to-stand test; HGS, hand-grip strength; FAS, fatigue assessment scale; PCFS, post-COVID-19 functional status; mMRC, modified Medical Research Council; Borg, modified Borg scale. Bold values denote *p* < 0.05; post hoc #, Pre vs. Post; ^*^, Pre vs. 1-year re-evaluation; &, Post vs. 1-year re-evaluation.

**Table 4:** Comparison of initial, final, and one-year re-evaluations in participants of the 36-session pulmonary rehabilitation group (*n* = 18).

**Variable**	**Pre PRP**	**Post PRP**	**1 year re-evaluation**	**Anova *p* value**
**Spirometrics parameters**				
FVC (L)	2.75 ± 0.75 (1.46–4.00)	3.00 ± 0.61 (2.15–3.86)	3.21 ± 0.62 (2.30–3.21)	**0.048**^**#**^ 0.014^*^
FVC (%Pred)	74.17 ± 20.88 (36.00–107.00)	83.56 ± 15.74 (53.00–107.00)	88.29 ± 13.78 (55.00–105.00)	**0.026**^**#**^ 0.01^*^
FEV_1_ (L)	2.22 ± 0.69 (0.40–3.31)	2.58 ± 0.57(1.82–3.52)	2.72 ± 1.97 (0.46–3.47)	**0.026**^**#**^ 0.032^*^
FEV_1_ (%Pred)	76.78 ± 25.22 (12.00–113.00)	88.22 ± 18.71 (60.00–133.00)	95.24 ± 14.79 (61.00–114.00)	**0.011# 0.022** ^*^
FEV_1_/FVC (%)	86.44 ± 8.84 (57.00–100.00)	85.59 ± 7.64(60.00–93.00)	85.35 ± 6.45 (69.00–99.00)	0.694
MIP (-cmH_2_O)	62.14 ± 31.03 (14.00–143.00)	74.72 ± 22.98 (14.50–104.10)	76.18 ± 21.76 (36.00–115.90)	**0.048**^**#**^ 0.040^*^
**Physical condition**				
6-MWD (m)	318.11 ± 114.68 (150.00–575.00)	471.22 ± 90.05 (311.00–620.00)	489.89 ± 102.12 (302.00–602.00)	**0.001** ^#, *^
STST (rpm)	25.22 ± 8.27 (0–34.00)	26.06 ± 7.64 (14.00–45.00)	24.28 ± 5.38 (11.00–33.00)	**0.001**^**#**^,^*^
HGS right (kg)	20.94 ± 18.59 (0–62.00)	30.44 ± 24.15 (0–84.00)	24.28 ± 48.63 (10.00–40.00)	**0.001**^**#**^ 0.042^*^
HGS left (kg)	17.33 ± 17.43 (0–60.00)	27.50 ± 22.32 (0–80.00)	23.61 ± 8.84 (8.00–36.00)	**0.001**^**#**^ 0.035^*^
**Fatigue, Functional status and dyspnea**				
Physical FAS (points)	14.33 ± 4.58 (7.00–22.00)	10.33 ± 3.85 (5.00 18.00)	12.56 ± 4.92 (5.00–22.00)	**0.001** ^**#**^
Mental FAS (points)	11.11 ± 3.74 (6.00–19.00)	8.83 ± 3.63 (5.00–17.00)	9.50 ± 4.24(5.00–18.00)	**0.025** ^**#**^
Total FAS (points)	25.44 ± 7.42 (13.00–39.00)	19.17 ± 19.17 (11.00–32.00)	22.06 ± 8.44 (11.00–40.00)	**0.001** ^**#**^
PCFS (points)	3.22 ± 0.87 (1.00–4.00)	1.50 ± 1.15 (0–3.00)	1.11 ± 1.02 (0–3.00)	**0.001** ^#, *^
mMRC (points)	2.22 ± 1.26 (0–4.00)	0.61 ± 0.97 (0–3.00)	0.61 ± 0.85(0–2.00)	**0.001** ^#, *^
Borg (points)	1.00 ± 1.13 (0–4.00)	0.18 ± 0.39 (0–1.00)	0.28 ± 0.66 (0–2.00)	**0.015**^**#**^ 0.034^*^
**Health-related quality of life**				
Physical functioning	36.94 ± 22.23 (0–80.00)	69.17 ± 19.03 (30.00–95.00)	76.39 ± 22.99 (25.00–100.00)	**0.001** ^#, *^
Role physical	8.33 ± 14.85 (0–50.00)	54.17 ± 40.12 (0–100.00)	51.39 ± 43.27 (0–100.00)	**0.001** ^#, *^
Bodily pain	36.80 ± 32.54 (0–100.00)	69.16 ± 27.27 (22.50–100.00)	60.97 ± 26.41 (10.00–100.00)	**0.001** ^**#**^
General health	47.78 ± 18.32 (5.00–90.00)	59.72 ± 21.79 (25.00–90.00)	51.39 ± 20.05 (15.00–90.00)	0.111
Vitality	37.50 ± 26.80 (0–85.00)	66.94 ± 22.95 (15.00–100.00)	65.28 ± 29.27 (25.00–100.00)	**0.001**^**#**^ 0.036^*^
Social functioning	60.41 ± 36.44 (0–100.00)	73.61 ± 29.67 (0–100.00)	84.72 ± 22.09 (37.50–100.00)	**0.023**
Role emotional	66.67 ± 48.50(0–100.00)	87.03 ± 32.61 (0–100.00)	66.66 ± 45.73 (0–100.00)	0.279
Mental health	23.61 ± 30.28 (0–100.00)	63.89 ± 35.58 (0–100.00)	84.72 ± 19.43 (50.00–100.00)	**0.001** ^#, *^

Data are presented as mean±standard deviation (minimummaximum). Pre RPR, before the pulmonary rehabilitation program; Post PRP, after the pulmonary rehabilitation program; FVC, forced vital capacity; FEV_1_, forced expiratory volume in the first second; MIP, maximum inspiratory pressure; cmH_2_O, centimeters of water; 6-MWD, 6-min walk test; STST, sit-to-stand test; HGS, hand-grip strength; FAS, fatigue assessment scale; PCFS, post-COVID-19 functional status; mMRC, modified Medical Research Council; Borg, modified Borg scale. Bold values denote *p* < 0.05; post hoc #, Pre vs. Post; ^*^, Pre vs. 1-year re-evaluation.

Regarding spirometric parameters, participants in the 12s group showed a significant increase in MIP between baseline and the completion of the 12 PRP sessions (*p* = 0.001). In terms of physical condition, an increase in 6-MWD distance was observed between baseline and the completion of the 12 PRP sessions (*p* = 0.001), as well as between baseline and the 1-year follow-up assessment (*p* = 0.001). The number of repetitions in the STST increased significantly between baseline and the completion of the PRP (*p* = 0.001), and between baseline and the 1-year reassessment (*p* = 0.001). Similarly, a significant increase was observed in right HGS between baseline and the completion of the PRP (*p* = 0.001), and between baseline and the 1-year follow-up (*p* = 0.007); the same trend was observed in left HGS values (Pre–Post, *p* = 0.001; Pre−1 year, *p* = 0.005). With respect to fatigue, functionality, and dyspnea, the physical FAS score decreased significantly between baseline and the end of the PRP (*p* = 0.001), as well as between the end of the PRP and the 1-year follow-up (*p* = 0.001). The mental FAS score decreased significantly after the PRP (*p* = 0.003) and remained stable at the 1-year follow-up. The total FAS score decreased significantly after the PRP (*p* = 0.001), and this result was maintained at the 1-year reassessment. In terms of functionality, the PCFS score decreased significantly between baseline and the end of the PRP (*p* = 0.001), and this improvement was sustained at the 1-year follow-up. For dyspnea, the mMRC score decreased significantly after the completion of the 12 PRP sessions (*p* = 0.001), and this reduction was maintained at the 1-year reassessment. Regarding HRQoL, the following items showed significant improvements after the PRP, which persisted at the 1-year follow-up: PF (*p* = 0.004; *p* = 0.001, respectively), PR (*p* = 0.010; *p* = 0.001, respectively), BP (*p* = 0.049; *p* = 0.006, respectively), VT (*p* = 0.012; *p* = 0.006, respectively), and mental health MH (*p* = 0.023; *p* = 0.001, respectively). Specifically, the MH item also showed a significant improvement between the end of the PRP and the 1-year reassessment (*p* = 0.037) (Table 2).

The comparison of assessments before and after 24 sessions of the PRP is presented in Table 3. Regarding spirometric parameters, there was a significant increase in the absolute value of FVC between baseline and the completion of the PRP (*p* = 0.05), as well as in MIP (*p* = 0.001). In terms of physical condition, a significant increase in 6-MWD distance was observed between baseline and after 24 PRP sessions (*p* = 0.001), and this improvement was sustained at the 1-year reassessment (*p* = 0.006). The number of repetitions in the STST increased significantly after the 24 PRP sessions (*p* = 0.001) and remained improved at the 1-year follow-up (*p* = 0.001). Additionally, both right and left HGS values increased significantly after the PRP (*p* = 0.011; *p* = 0.048, respectively). With respect to fatigue, functionality, and dyspnea, the physical FAS score decreased significantly between baseline and the 1-year reassessment (*p* = 0.001), as did the mental FAS score (*p* = 0.026) and the total FAS score (*p* = 0.002). In terms of functionality, the PCFS score decreased significantly between baseline and the completion of the PRP (*p* = 0.001), and this improvement was maintained at the 1-year follow-up (*p* = 0.001). For dyspnea, the mMRC score decreased significantly between baseline and the end of the PRP (*p* = 0.001), and this reduction was sustained at the 1-year reassessment (*p* = 0.001). Regarding HRQoL, significant improvements were observed between baseline and the end of the PRP, which were maintained at the 1-year reassessment, in the following items: PF (*p* = 0.039; *p* = 0.004, respectively), VT (*p* = 0.005; *p* = 0.004, respectively), SF (*p* = 0.011; *p* = 0.042, respectively), and MH (*p* = 0.001; *p* = 0.001, respectively) (Table 3).

In relation to the participants in the 36s group, spirometric parameters showed a significant increase between the baseline evaluation and the end of the 36 PRP sessions. Changes that were maintained at the 1-year re-evaluation in the variables FVC (*p* = 0.048; *p* = 0.014, respectively), FEV_1_ (*p* = 0.026; *p* = 0.032, respectively), and PIM (*p* = 0.048; *p* = 0.040, respectively). In terms of physical fitness, the distance covered in the 6-MWD increased significantly after the PRP (*p* = 0.001) and 1 year after the re-evaluation (*p* = 0.001) in relation to the baseline evaluation. The number of repetitions in the STST increased after the PRP (*p* = 0.001) and 1 year after the re-evaluation (*p* = 0.001). In the right (p = 0.001) and left (p = 0.048) HGS, there was a significant increase in strength after completing PRP compared to the baseline assessment. Regarding fatigue, functionality, and quality of life, the physical (p = 0.001), mental (*p* = 0.026), and total (*p* = 0.002) FAS scores decreased significantly between the baseline assessment and the 1-year re-assessment. The PCFS score decreased significantly after the end of the PRP, a value that was maintained at the 1-year reassessment (*p* = 0.001; *p* = 0.001, respectively). Dyspnea measured with the mMRC decreased significantly after the 36 PRP sessions (*p* = 0.001), a result that was maintained at the 1-year reassessment (*p* = 0.001). In HRQoL, a significant improvement was observed at the end of the PRP, which was maintained at the 1-year reassessment, in the following items: PF (*p* = 0.039; *p* = 0.004, respectively), VT (*p* = 0.005; *p* = 0.004, respectively), SF (*p* = 0.011; *p* = 0.042, respectively), and MH (*p* = 0.001; *p* = 0.001, respectively) (Table 4).

### Pulmonary rehabilitation, final results at 1 year per group

The results of the 1-year follow-up assessment after completion of the PRP across the three groups are presented in Table 5. Regarding fatigue and functionality, the physical FAS score was significantly lower in the 12s group compared to the 36s group (*p* = 0.008); likewise, the total FAS score was significantly lower in the 12s group compared to the 36s group (*p* = 0.008). In terms of functionality, the PCFS score was significantly lower in the 12s group compared to the 24s group (*p* = 0.037). On the other hand, in HRQoL, the BP item score was significantly higher in the 12s group compared to the 24s group (*p* = 0.008). Finally, the prevalence of diabetes mellitus remained significantly higher in the 12s group (Table 5).

**Table 5:** Comparison of the final evaluation of the participants, divided into groups according to the number of sessions.

**Variable**	**12s Group**	**24s Group**	**36s Group**	***p*-value**
**General characteristics**				
N/percentage (Female/Male)	25/42% (9/16)	17/28% (6/11)	18/30% (6/12)	0.984
Age (Years)	60 ± 13 (19.00–78.00)	63 ± 7 (52.00–78.00)	58 ± 9 (46.00–78.00)	0.376
Weight (kg)	88.07 ± 15.53 (64.00–116.00)	89.47 ± 17.56 (70.00–130.00)	90.15 ± 18.50 (70.00–138.00)	0.920
Height (m)	1.63 ± 0.07 (1.46–1.74)	1.64 ± 0.07 (1.50–1.79)	1.63 ± 0.07 (1.45–1.74)	0.648
BMI (kg/m^2^)	33.27 ± 6.04 (24.70–44.60)	33.32 ± 7.22 (24.40–49.00)	31.57 ± 5.70 (24.60–42.30)	0.699
IMV (days)	20.68 ± 12.28 (3.00–43.00)	27.06 ± 21.08 (7.00–75.00)	35.94 ± 32.10 (4.00–138.00)	0.095
Worker (Yes/No)	17/8	11/6	12/6	-
**Spirometrics parameters**				
FVC (L)	3.47 ± 0.82 (1.65–5.07)	3.05 ± 0.83 (1.51–4.31)	3.21 ± 0.62 (2.30–3.21)	0.233
FVC (%Pred)	96.80 ± 15.95 (63.00–130.00)	87.38 ± 17.62 (57.00–124.00)	88.29 ± 13.78 (55.00–105.00)	0.112
FEV_1_ (L)	2.85 ± 0.67(1.41–4.15)	2.53 ± 0.76 (1.00–3.72)	2.72 ± 1.97 (0.46–3.47)	0.308
FEV_1_ (%Pred)	101.52 ± 17.51 (65.00–132.00)	92.31 ± 19.61 (48.00–130.00)	95.24 ± 14.79 (61.00–114.00)	0.230
FEV_1_/FVC (%)	82.28 ± 4.07 (57.00–100.00)	82.06 ± 5.97 (66.00–90.00)	85.35 ± 6.45 (69.00–99.00)	0.136
MIP (-cmH_2_O)	73.88 ± 19.03 (39.00–111.10)	71.85 ± 26.50 (27.80–109.90)	76.18 ± 21.76 (36.00–115.90)	0.852
**Physical condition**				
6-MWD (m)	516.72 ± 94.35 (275.00–620.00)	481.07 ± 105.22 (193.00–603.00)	489.89 ± 102.12 (302.00–602.00)	0.502
STST (rpm)	26.96 ± 6.92 (12.00–41.00)	25.21 ± 5.87 (8.00–33.00)	24.28 ± 5.38(11.00–33.00)	0.330
HGS right (kg)	25.44 ± 8.13 (10.00–40.00)	22.19 ± 11.32 (5.00–41.00)	24.28 ± 48.63 (10.00–40.00)	0.552
HGS left (kg)	23.60 ± 7.62(9.00–35.00)	19.69 ± 11.31 (0–37.00)	23.61 ± 8.84 (8.00–36.00)	0.347
**Fatigue, functional status and dyspnea**				
Physical FAS (points)	8.40 ± 3.45 (5.00–18.00)	10.53 ± 4.71 (6.00–19.00)	12.56 ± 4.92 (5.00–22.00)	**0.008** ^*^
Mental FAS (points)	7.72 ± 3.06 (5.00–19.00)	9.88 ± 4.35 (5.00–18.00)	9.50 ± 4.24 (5.00–18.00)	0.149
Total FAS (points)	16.12 ± 6.16 (10.00–36.00)	20.41 ± 8.39 (11.00–37.00)	22.06 ± 8.44 (11.00–40.00)	**0.008** ^*^
PCFS (points)	0.68 ± 0.74 (0–2.00)	1.35 ± 0.70 (0–3.00)	1.11 ± 1.02 (0–3.00)	**0.037** ^**#**^
mMRC (points)	0.28 ± 0.61 (0–2.00)	0.53 ± 0.80 (0–3.00)	0.61 ± 0.85 (0–2.00)	0.317
Borg (points)	0.12 ± 0.33 (0–1.00)	0.29 ± 0.77 (0–3.00)	0.28 ± 0.66 (0–2.00)	0.563
**Health-related quality of life**				
Physical functioning	79.80 ± 20.69 (20.00–100.00)	75.63 ± 25.55 (0–100.00)	76.39 ± 22.99 (25.00–100.00)	0.818
Role physical	67.00 ± 44.32 (0–100.00)	40.63 ± 36.37 (0–100.00)	51.39 ± 43.27 (0–100.00)	0.142
Bodily pain	71.56 ± 23.53 (25.00–100.00)	47.18 ± 21.86 (10.00–80.00)	60.97 ± 26.41 (10.00–100.00)	**0.008** ^**#**^
General health	62.71 ± 19.44 (15.00–95.00)	48.75 ± 21.79 (5.00–90.00)	51.39 ± 20.05(15.00–90.00)	0.072
Vitality	75.21 ± 20.02 (30.00–100.00)	61.88 ± 25.68 (10.00–100.00)	65.28 ± 29.27 (25.00–100.00)	0.210
Social functioning	80.67 ± 16.95 (48.00–100.00)	69.53 ± 31.94 (12.50–100.00)	84.72 ± 22.09 (37.50–100.00)	0.082
Role emotional	75.00 ± 41.99 (0–100.00)	47.91 ± 47.09 (0–100.00)	66.66 ± 45.73 (0–100.00)	0.057
Mental health	80.21 ± 23.28 (50.00–100.00)	71.88 ± 23.93 (25.00–100.00)	84.72 ± 19.43 (50.00–100.00)	0.248
**Medical history**				
Obesity (*n*/%)	14 (56.00%)	7 (41.17%)	8 (44.44%)	0.814
HBP(*n*/%)	16 (64.00%)	14 (82.35%)	10 (55.55%)	0.497
DM (*n*/%)	17 (68.00%)	8 (47 %)	6 (33.33%)	**0.040** ^*^

Data are presented as mean ± standard deviation (minimum-maximum). Bold values denote *p* < 0.05; 12s, group “12 sessions”; 24s, group “24 sessions”; 36s, group “36 sessions”; N, number of participants; BMI, body mass index; IMV, invasive mechanical ventilation; FVC, forced vital capacity; FEV_1_, forced expiratory volume in the first second; MIP, maximum inspiratory pressure; cmH_2_O, centimeters of water; 6-MWD, 6-min walk distance; STST, sit-to-stand test; HGS, hand-grip strength; FAS, fatigue assessment scale; PCFS, post-COVID-19 functional status; mMRC, modified Medical Research Council; Borg, modified Borg scale; %, Percentage; DM, diabetes mellitus; HBP, high blood pressure;; #, 12s vs. 24 s; ^*^, 12s vs. 36s.

## Discussion

This study assessed the long-term effects (~1 year) of an individualized PRP (12s vs. 24s vs. 24s) in patients with COVID-19 connected to IMV. In this regard, the main findings of this study indicated that: (i) after 1 year of individualized PRP, there were no significant differences in spirometric variables, exercise capacity, or quality of life (except the variable BP), regardless of the number of sessions performed; (ii) most of these variables showed significant changes between the initial and 1-year evaluations, except for MH in the 12-session group, which also showed significant differences between the post-PRP and 1-year evaluations. These results are in partial agreement with those reported by O'Brien et al. (15), who observed that survivors of COVID-19 hospitalization report persistent symptoms, particularly fatigue, shortness of breath, low HRQoL scores, and suboptimal exercise levels. Here, the evidence indicates that patients with sequelae of COVID-19 require a multidisciplinary intervention involving long-term follow-up of the evolution of the symptoms to identify possible complications and propose clinical interventions based on physical exercise (27). However, studies on this subject are scarce.

Concerning exercise capacity, the results of this study indicate that there was a significant increase in the distance covered in the 6-MWD, the number of repetitions in the STST, and HGS after the PRP in the three groups (Tables 2–4), which was maintained at 1 year of re-evaluation. In this context, Peball et al. (28) evaluated the long-term consequences (1 year) after COVID-19, specifically physical disorders that could affect physical recovery and quality of life. One of their main outcomes was the significant decrease in the distance traveled in 6-MWD in patients with severe COVID-19 compared to those with mild COVID-19 (severe: 547.2 m vs. mild: 606.3 m; *p* = 0.044). However, of the total sample, only 23% (16 participants) received rehabilitation, unlike the sample in this study, all of whom participated in a PRP. Therefore, it is important to highlight that (i) by classification, all patients in this study presented severe COVID-19, (ii) in addition to the distance covered, we can complement the analysis of exercise capacity with the STST and HGS outcomes, and (iii) after the PRP period there were no significant differences between the groups in the three evaluations. Thus, the strategy of customizing the workload in conjunction with the number of sessions proved useful.

Regarding fatigue and functionality, the baseline assessment showed that patients in the 24s and 36s groups started with a higher level of fatigue than patients in the 12s group (Table 1). Although the values improved significantly after the PRP, the differences between the groups remained the same at the 1-year evaluation. On the other hand, all three groups experienced a significant decrease in the PCFS scale score, which did not change at the 1-year re-evaluation. Considering fatigue, O'Brien et al. (15) examined self-reported physical recovery and well-being in patients with COVID-19 at 10 weeks, 6 months, and 1 year after hospital discharge. This group observed a significant improvement in 6-MWD with time (*F* = 10.3; *p* < 0.001) from 365 (±209) m at T1 to 447(±85) m at T3; however, this outcome remained below the reference values. In addition, fatigue was the most frequently reported symptom at T1 (40%) and T2 (49%) (15). On the other hand, memory and/or concentration problems were reported more frequently in T3 (49%). Betscharta et al. (13) described the physical performance and HRQoL of a Swiss cohort recovering from COVID-19 1 year after hospitalization. They were evaluated three times: hospital discharge, 3 months, and 12 months post-admission. Functionality, measured through the PCFS scale, revealed that, at 1 year, 12 of 41 participants still perceived limitations related to COVID-19 in daily life (PCFS: 2 points = 10; 3 points = 2) (13). Although there are agreements in fatigue perception, the behaviors are different because, after the PRP, there was a significant improvement, which, in the psychological fatigue item, declines at 1 year. In contrast, the improvement in functionality was maintained at the 1-year re-evaluation. Regarding these findings, while we acknowledge the merit of customizing the workloads, we also perceive a deficiency in the absence of a psychologist and a nutritionist on the PRP team. These specialists could have provided valuable support and further improved the outcomes of the suggested training program.

At the 1-year evaluation, the HRQoL outcomes showed a lower score in the 24s and 36s groups compared to the 12s group. However, the 1-year re-evaluation showed that bodily pain was the problem that persisted in the 24-session group (Table 5). In this context, Bek et al. (29) concluded that up to 12 months after hospitalization for COVID-19, HRQoL remained reduced compared to the general population, symptoms persisted, and a considerable proportion of patients reported incomplete recovery. Similarly, Betschart et al. (13) observed that 12 of 41 participants still perceived symptoms of moderate to severe bodily pain and discomfort, and 13 of 41 patients had mild to severe symptoms of anxiety and depression. Conversely, O'Brien et al. (15) reported that the SF-36 scores in patients with COVID-19, 1 year after hospital discharge, did not change significantly in any domain, in addition to being below the population norms in the domains of physical functioning, energy/vitality, limitations due to physical problems and general health. These outcomes contrast with those of the present study, where, except for bodily pain, the remaining HRQoL variables improved. This could be due to our participants undergoing a period of respiratory rehabilitation, which was adapted in terms of workload and number of sessions.

Another important point to highlight is that regardless of the number of sessions, the three groups showed a significant increase in the outcomes of the physical performance tests, which was maintained after 1 year of re-evaluation. At the same time, there were no significant differences when comparing these same outcomes among the different groups (Table 5). In this context, Dun et al. (30) investigated the effects of pulmonary rehabilitation on immune and exercise capacity 6 months after hospitalization for COVID-19. Their main results showed that a PRP significantly increased the distance traveled in the 6-MWD compared to the control group [unadjusted, 194 (167–221) m, *p* < 0.001; adjusted, 123 (68–181)] m, *p* < 0.001). Specifically, the percentage change in distance traveled in 6-MWD was significantly higher in the group with more sessions (≥ 17 sessions) than the group with fewer sessions ( ≤ 5 sessions) and the control group [165 (101–229) m vs. 77 (34–120) m, *p* = 0.009, respectively] (30). In contrast, the current study generated significant changes in physical capacity regardless of the number of sessions, which could be because (i) the present study proposed delivering a standard number of sessions in consideration of the initial evaluation of the patients, (ii) the patients attended all their sessions, with the possibility of rescheduling or recovering sessions, and (iii) the workload was personalized and re-evaluated every 2 weeks for both aerobic and strength exercise. Collectively, these measures effectively addressed the specific needs of each patient.

This research has limitations that are important to point out: (i) a limitation of this study is that it is not a randomized clinical trial, (ii) losses to 1 year follow-up were documented (statistical power calculated at 0.67), (iii) the PRP did not have psychological support, which could have benefitted the treatment in the management of the perception of fatigue and bodily pain, and (iv) there was no record of the activities performed by each participant in the period between the end of the PRP and the 1-year evaluation.

This study concluded that the benefits achieved in spirometric values, aerobic capacity, and muscle strength after individualized PRP were maintained 1 year later. There were no differences among the three groups (12s vs. 24s vs. 36s) at the 1-year re-evaluation. Therefore, irrespective of the initial severity on admission, conducting a personalized PRP treatment will sustain the achieved results 1 year later.

## Data Availability

The original contributions presented in the study are included in the article/supplementary material, further inquiries can be directed to the corresponding author.
